# Non-Covalent Cross-Linking Hydrogel: A New Method for Visceral Hemostasis

**DOI:** 10.3390/gels10050326

**Published:** 2024-05-10

**Authors:** Chenyu Zhao, Han Wang, Xue Sun, Ying Liu, Jingjing Chen, Jiaqi Li, Fanshan Qiu, Qianqian Han

**Affiliations:** 1 National Institutes for Food and Drug Control, Beijing 100050, China; a_zhaochenyu@163.com (C.Z.); wanghan926926@126.com (H.W.); sunxue@nifdc.org.cn (X.S.); chenjing151@126.com (J.C.); cpuljq1996@126.com (J.L.); 15273390558@163.com (F.Q.); 2 Beijing Laboratory of Biomedical Materials, Beijing University of Chemical Technology, Beijing 100029, China; liuying1703@126.com; 3 Department of Pharmaceutics, China Pharmaceutical University, Nanjing 211198, China

**Keywords:** bioadhesive, hemostasis, Tannic acid, biodegradability, biocompatibility

## Abstract

Excessive blood loss could lead to pathological conditions such as tissue necrosis, organ failure, and death. The limitations of recently developed hemostatic approaches, such as their low mechanical strength, inadequate wet tissue adhesion, and weak hemostatic activity, pose challenges for their application in controlling visceral bleeding. In this study, a novel hydrogel (CT) made of collagen and tannic acid (TA) was proposed. By altering the proportions between the two materials, the mechanical properties, adhesion, and coagulation ability were evaluated. Compared to commercial hydrogels, this hydrogel has shown reduced blood loss and shorter hemostatic time in rat hepatic and cardiac bleeding models. This was explained by the hydrogel’s natural hemostatic properties and the significant benefits of wound closure in a moist environment. Better biodegradability was achieved through the non-covalent connection between tannic acid and collagen, allowing for hemostasis without hindering subsequent tissue repair. Therefore, this hydrogel is a new method for visceral hemostasis that offers significant advantages in treating acute wounds and controlling major bleeding. And the production method is simple and efficient, which facilitates its translation to clinical applications.

## 1. Introduction

Millions of surgeries are carried out, ranging from minor wounds to major emergency procedures. Adequate management of emergency hemostasis during surgery and proper wound closure following the procedure are essential to prevent issues like wound infection and postoperative bleeding [1]. Excessive blood loss could lead to pathological conditions such as tissue necrosis, organ failure, and death [2]. Furthermore, some patients may be at a higher risk of bleeding due to drug-induced side effects or congenital or acquired diseases [3]. Clinical treatments typically involve the use of sutures and staples to close wounds. However, these methods have several disadvantages, including the risk of secondary infection and difficulty in manipulation [4,5]. In general, it is difficult for endogenous hemostatic mechanisms to halt visceral bleeding [6,7,8]. The ideal hemostatic material should promptly seal the bleeding wound upon contact with the bleeding site and achieve both physical and chemical hemostasis [9,10].

Materials with hemostatic properties can be divided into three categories based on their mechanisms. The first type includes pro-coagulant factors such as thrombin or coagulation factors, which aid in stopping bleeding quickly in wounds, such as fibrin [11,12]. However, this type carries the risk of releasing hemostatic agents into the vascular system, which can cause thrombosis in downstream blood vessels [13]. The second type recruits coagulant substances around the wound through chemical or physical action, achieving rapid hemostasis. These substances, such as zeolite, kaolin, and chitosan [14], are effective in stopping bleeding but may not be suitable for deep wounds due to their irregular shape and low elasticity. The third type aims to achieve hemostasis by sealing the wound, and some adhesives, such as α-cyanoacrylate [15,16], can achieve this without activating the coagulation cascade. However, they have several disadvantages, including the curing and exothermic stimulation of the wound [17], difficulty in peeling off [18], and toxic degradation products. Scientists have developed several effective bioadhesives that can be applied rapidly to achieve hemostasis, compensating for the limitations of commercial hemostats. Specifically, certain hemostatic materials can recruit blood components at the site of bleeding [19], facilitate the coagulation cascade, or serve as a physical barrier. For instance, Hyuwoo [20] was inspired by barnacle glue to investigate a hemostatic material with a potent hemostatic effect. This material embeds a lipid-rich matrix in an adhesive protein, which is beneficial for patients with coagulation disorders or those taking anticoagulant drugs. Mooney [21] studied a novel adhesive inspired by the shielding mucus generated by slugs and used it successfully to heal a pig’s heart. However, producing these hydrogels is a challenging, costly, and time-consuming process.

For visceral hemorrhage, it is necessary to ensure that strong adhesion is maintained under the impact of continuous blood flow to achieve wound closure. The discovery of catechol-based adhesives in wet adhesion provides valuable biomimetic inspiration for developing a range of hydrogels suitable for use in aquatic environments. The actual implementation of these biomimetic hydrogels may be hindered by several issues, such as limited adhesion strength, prolonged curing time, and the release of hazardous organic solvents. Strong adhesion in water, especially in the blood environment, is still lacking despite the description and demonstration of several dopamine-based hydrogels that adhere to various material surfaces. Tannic acid (TA) is a natural polyphenol that possesses unique biological activities, including antioxidant, antimicrobial, and procoagulant properties [22,23]. Tannic acid (TA) is rich in phenolic hydroxyl groups (-OH), which exhibit a strong adhesion capacity [24]. TA can react with blood proteins such as serum albumin, globulin, and coagulation factors, thereby promoting blood coagulation [25]. Collagen is a significant protein in the extracellular matrix due to its low immunogenicity and good biocompatibility [26]. It is a potent platelet activator that promotes thrombin production by interacting with platelet receptors (α2β1, glycoprotein VI, and von Willebrand Factor) [27]. Additionally, it activates the intrinsic coagulation pathway by binding to coagulation factor XII [28]. Collagen-based hydrogels have been extensively studied in the fields of tissue engineering and biomedicine [26,29,30]. However, collagen’s weak mechanical properties limit its widespread use [31]. To solve this problem, other biomaterials, such as hyaluronic acid, chitosan, and alginate, have been added to collagen.

**Scheme 1:**
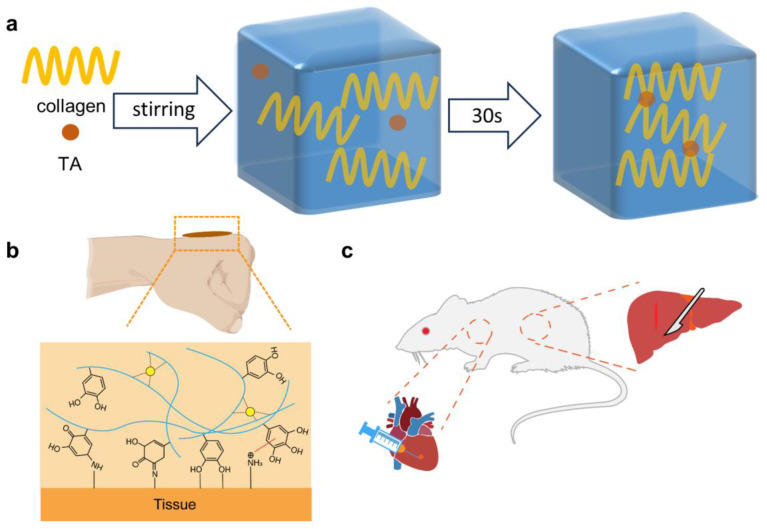
The illustration of (**a**) the preparation and (**b**) cross-linking network structure of CT hydrogel for (**c**) heart and liver hemostasis.

This paper investigated a hydrogel called CT, which was made of tannic acid and collagen. Tannins bind to collagen through hydrogen bonding to create a stable non-covalent cross-linked network (Scheme 1). The study explored the mechanical properties, adhesion properties, biocompatibility, and in vitro procoagulant coagulation properties of the hydrogel, and optimized its formulation. Additionally, the study compared the in vivo hemostatic properties of the hydrogel with those of cyanoacrylates in rat liver and heart hemorrhage models. Finally, the hydrogel was implanted subcutaneously in the backs of rats to study its in vivo degradation properties and tissue characteristics. The composite bioadhesive is simple to manipulate and cost-effective, and exhibits strong adhesion properties in humid environments. Furthermore, it exhibits good biocompatibility and efficient visceral hemostasis.

## 2. Results and Discussion

### 2.1. Characterization of the Hydrogels

**Figure 1:**
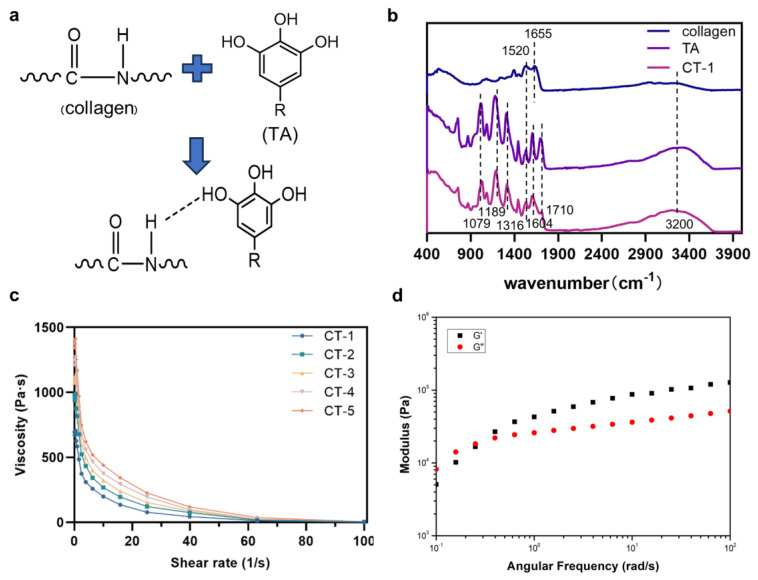
(**a**) Cross-linking structure; (**b**) FT-IR spectroscopy of the hydrogel, collagen, and tannic acid; (**c**) the viscosity test of hydrogels with different proportions; (**d**) the G′ and G″ of hydrogel CT-1.

Hydrogels composed of tannic acid (TA) and collagen were prepared using a physical mixing method. Due to the weak mechanical properties of collagen, a lower concentration is not sufficient to achieve cross-linking. In order to form a stable network structure, a collagen concentration of 10 wt% was chosen through preliminary experiments [28]. TA solutions with varying concentrations were added to the collagen solution and stirred thoroughly. The mass ratios of tannic acid to collagen were 1:1, 1:2, 1:3, 1:4, and 1:5, respectively, and were labeled as CT-1, CT-2, CT-3, CT-4, and CT-5. TA interacts with collagen via hydrogen bonding to create a tightly cross-linked structure (Figure 1a). TA acts as a crosslinker for the overall matrix and matrix-tissue bonding of the system, while collagen provides the matrix network.

The structure of the hydrogels was characterized by FT-IR spectroscopy (Figure 1b). The Ar–OH stretching vibration of tannic acid was observed between 3500 cm^−1^ and 3000 cm^−1^, showing a broad and intense spectral band. Additionally, the C=O stretching vibration, C=C stretching vibration, and C–C stretching vibration of aromatic molecules were observed at 1710 cm^−1^, 1604 cm^−1^, and 1532 cm^−1^, respectively. The O–H bending vibration of ether was observed at 1316 cm^−1^, while the C–O stretching vibration and C–O stretching vibration of polyol were observed at 1189 cm^−1^ and 1079 cm^−1^, respectively. These were the characteristic peaks of tannic acid. In the collagen spectrum, the peaks at 1655 cm^−1^ and 1520 cm^−1^ correspond to the C=O stretching vibration of protein and the N–H bending vibration of amide, respectively. The hydrogel contained all the characteristic peaks of collagen and tannic acid, with no new peaks appearing. Only the positions of individual peaks shifted, indicating that there was no covalent bond formation between the two components, but rather non-covalent interactions. The hydroxyl and amine groups of the collagen side chain can formed hydrogen bonds with the numerous phenolic hydroxyl groups present in tannic acid (Figure S1), leading to physical cross-linking. The presence of these hydrogen bonds was crucial for adhesive generation. The FT-IR of the hydrogel in the wet state was tested (Figure S2), and only the positions of individual peaks shifted, indicating that the hydrogel structure was not altered in the wet state. Interactions such as hydrogen bonding between water and the hydrogel resulted in the attenuation of some of the characteristic peaks of the hydrogel.

Rheological tests were also used to investigate the mechanical properties of the hydrogel. As shown in Figure 1c, the viscosity of the hydrogel decreased with an increasing shear rate, indicating the shear thinning behavior of the hydrogels. The storage modulus (G′) and loss modulus (G″) of the hydrogel were measured through an angular frequency sweep (Figure 1d). Across the frequency range (1–100 rad/s), the G′ of the hydrogel was higher than the G″, indicating that the hydrogels were stable and displayed predominantly elastic behavior. Both the G′ and G″ of the hydrogels showed an increasing trend with increasing TA content (Figure S3). In addition, the G′ of the hydrogels gradually increased with the increasing concentration of TA. This may be attributed to the fact that TA increased the cross-linking density of the hydrogels and induced strong interactions between the polymers [32,33]. Therefore, the CT-5 with the highest TA content exhibited the best mechanical properties.

**Figure 2:**
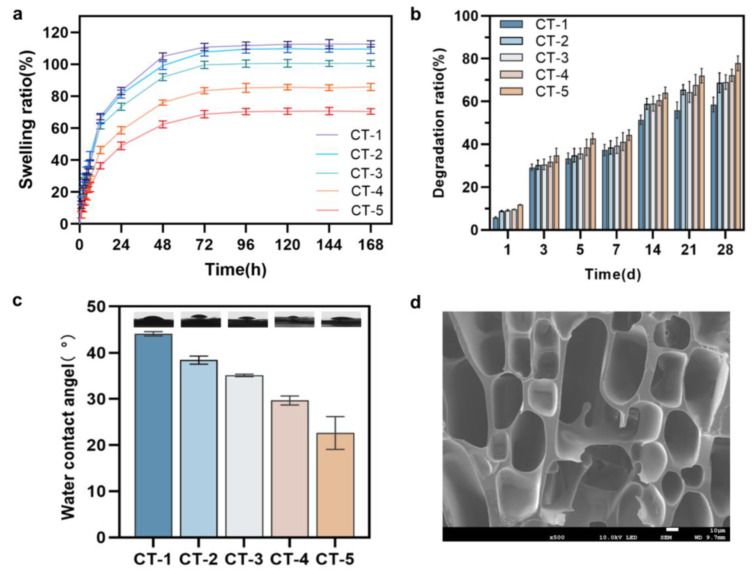
(**a**) Swelling ratio of the hydrogel; (**b**) In vitro degradation of the hydrogel; (**c**) Water contact angle test of the hydrogel; (**d**) SEM image of the hydrogel CT-1.

The swelling and degradation behavior of hydrogels is essential for tissue regeneration, hydrogel stability, and the absorption of interstitial fluid [34]. According to the swelling curve (Figure 2a), the hydrogels took 72 h to reach swelling equilibrium. CT-1 and CT-2 exhibited the highest swelling rate, exceeding 100%, while CT-5 showed the least amount of swelling, approximately 60%. A hydrogel’s ability to promote blood coagulation, enrich red blood cells, and draw blood from the wound site depends on a specific rate of swelling [35]. To monitor the stability of the hydrogel in PBS at 37 °C, an in vitro degradation experiment (Figure 2b) was conducted. The results demonstrated its potential to degrade in PBS, with a degradation rate of approximately 40% in 7 days and 70% in 28 days. For wound and tissue healing, it is advantageous for the material to be degradable, as it can be used in visceral hemostasis.

The hydrophilic and hydrophobic qualities of hydrogels were tested by examining its water contact angle (Figure 2c) since it is primarily used in humid environments. All angles were found to be less than 90°, demonstrating their hydrophilicity and ability to support adhesion in a moist state. The water contact angle increased with the rising TA content, indicating greater hydrophilicity.

SEM was used to observe the morphological structure of the hydrogels (Figure 2d). As the fraction of TA increased, the porosity of the hydrogel also increased, with a pore size of approximately 3 μm (Figure S4). This porous structure facilitates the adsorption of red blood cells and platelets on the surface of bleeding tissue, leading to rapid hemostasis [35].

### 2.2. Bio-Adhesive of the Hydrogel

**Figure 3:**
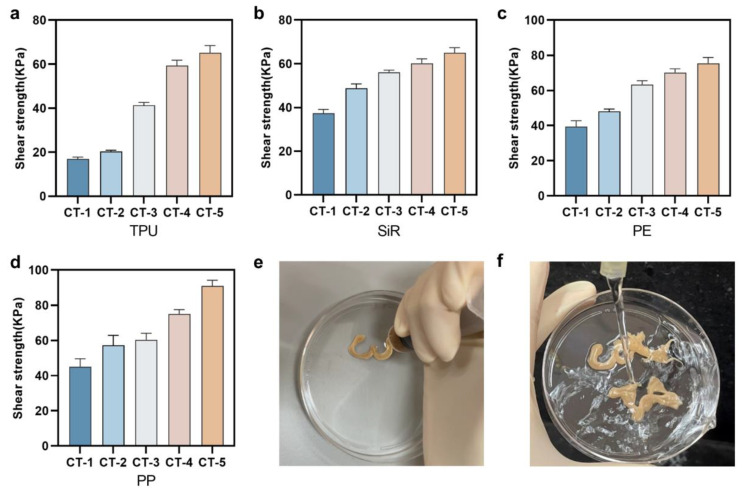
Shear adhesion of (**a**) TPU, (**b**) SiR, (**c**) PE, (**d**) PP; (**e**) injectability of the hydrogel; (**f**) wet adhesion of the hydrogel.

As shown in Figure 3a–d and Figure S5, the hydrogels exhibited good adhesion to various substrates, such as polytetrafluoroethylene (PTFE), wood, steel, glass, and thermoplastic polyurethanes (TPU). The results showed that the adhesive strength increased from 45.11 ± 4.48 kPa to 90.99 ± 3.22 kPa as the TA content increased. As shown in Figure 3e,f, the hydrogels were extruded through a syringe to form the letters “COL-TA” and adhered to the plate immediately. This adhesion persisted even as water flowed over it.

**Figure 4:**
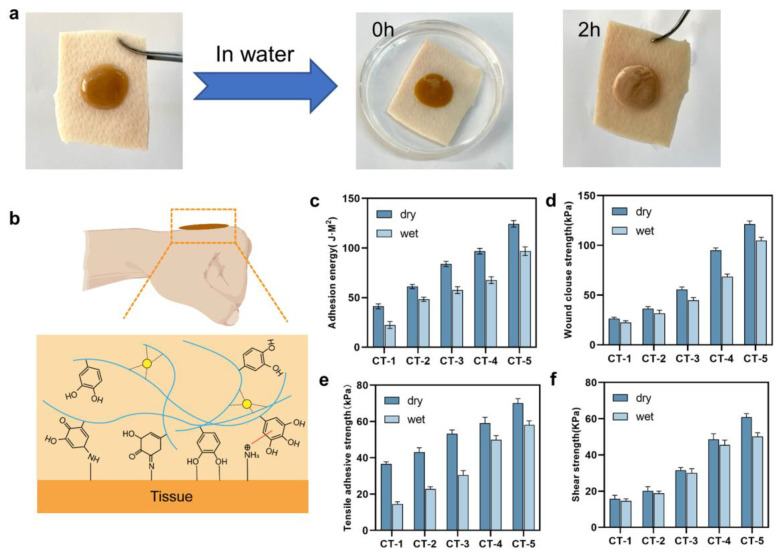
(**a**) Bioadhesion of the hydrogel; (**b**) adhesion mechanism on tissue; (**c**) peel strength, (**d**) wound-closure test, (**e**) lap shear test of the hydrogel, (**f**) tensile strength.

One of the most crucial characteristics of tissue adhesives is their ability to adhere to tissue. As shown in Figure 4a, the hydrogel could firmly adhere to porcine skin and remained attached even after 2 hours in water, indicating its strong adhesion to wet tissue surfaces. Several factors contribute to the hydrogel’s excellent adhesion and durability. As shown in Figure 4b, adhesion between TA and the skin tissue surface was achieved with a variety of forces, including hydrogen bonding, cationic interaction, π interaction, and covalent bonding (Michael addition and Schiff base reactions) [36]. The abundance of hydroxyl groups in TA gives it its unique stickiness (Figure S1). Additionally, TA functions as a cross-linking agent through hydrogen bonding, further strengthening the multiple cross-linking network. It is clear that TA plays a crucial role in enhancing bonding and performance. To assess the tissue adhesion strength of the hydrogel, four experiments were conducted on porcine skin tissues (Figure S6). As shown in Figure 4c–f, the adhesion strengths of the hydrogels to porcine skin increased proportionally with TA content in both dry and wet conditions. The CT-1 exhibited the lowest adhesion strength among the tested formulations (14.45 ± 1.35 kPa), whereas CT-5 demonstrated the highest adhesion strength (104.9 ± 3.30 kPa) in wet conditions. The adhesion strengths of the hydrogels were higher than those of commercially available fibrin glue, which has a strength of 4.58 ± 1.57 kPa. Additionally, the hydrogel demonstrated good tissue adhesion to rat viscera, including the heart, liver, spleen, lung, kidney, and stomach (Figure S7). These results suggest that the hydrogel is an effective visceral hemostatic agent with the necessary tissue adhesion strength.

Based on the experimental results presented above, CT-3, CT-4, and CT-5 were chosen for follow-up experiments because of their hydrogel stability and adhesion strength, even in wet environments.

### 2.3. Hemocompatibility and In Vitro Pro-Coagulant Performance of the Hydrogel

**Figure 5:**
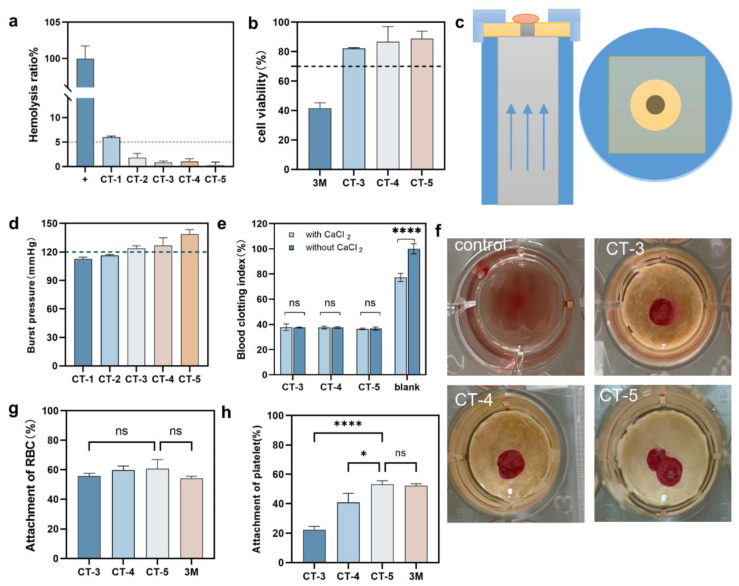
(**a**) Hemolysis experiments of the hydrogel; (**b**) cytotoxicity of the hydrogel; (**c**) schematic diagram of the burst pressure test device; (**d**) burst pressure test of the hydrogel; (**e**) BCI of the hydrogel; (**f**) photograph of BCI between the control group and the sample group; (**g**) red blood cell attachment; (**h**) platelet adhesion assay. (* *p* < 0.05, **** *p* < 0.0001, ns *p* > 0.05).

Excellent hemostatic materials must be hemocompatible and cytocompatible [37]. First, the hemocompatibility of the CT hydrogel was evaluated through hemolysis assays. As shown in Figure 5a, the hemolysis ratios of all hydrogel groups were lower than 5%, meeting the international standard for biomaterials [38]. Furthermore, the cytocompatibility of the CT hydrogel was assessed. As shown in Figure 5b, the activity of L929 cells in all hydrogel groups was above 80%, which complies with the international standard for biomaterials. In contrast, the commercial adhesive group (3M) resulted in extensive cell death. The main components of the hydrogels were collagen and TA, which are natural biopolymers with excellent biocompatibility. Water was the only solvent used during adhesive preparation. These factors ensured that the hydrogels exhibited excellent biocompatibility. Hemostatic materials must possess adequate tissue adhesion to withstand blood flow and endure stress in the dynamic physiological environment of the body. The burst pressure test evaluated the hydrogel’s capacity to endure the extra pressure from blood flow during bleeding, utilizing the device depicted in Figure 5c. As shown in Figure 5d, the CT-3, CT-4, and CT-5 hydrogels exhibited a burst pressure exceeding 120 mmHg, indicating their capacity to withstand the typical systolic arterial pressure observed in humans, and their suitability for visceral hemostasis.

The blood clotting index (BCI) is a crucial indicator for evaluating the procoagulant capacity of hemostatic materials in vitro [39]. Anticoagulated whole blood with added Ca^2+^ was placed in full contact with the hydrogel for 5 min. Subsequently, the uncoagulated blood was lysed with purified water. The procoagulant ability of the hydrogel was assessed by measuring the absorbance of the supernatant [40]. As shown in Figure 5f, the transparency of the solution in the hydrogel group was significantly higher than that of the control group, indicating that their blood had coagulated. As shown in Figure 5e, the BCI values of the hydrogels were significantly lower than the control, indicating that the hydrogel had a significant hemostatic advantage. Furthermore, the hydrogels demonstrated similar BCI values with or without the addition of exogenous Ca^2+^. This suggests that the procoagulant ability of the hydrogel is most likely through the activation of the contact/intrinsic pathway of coagulation through the activation of FXII [41]. This process is not calcium-dependent and can be elicited by collagen [42].

Proper hemostasis behaviors require stimulating platelet accumulation and promoting thrombosis in injured tissues [43]. To evaluate the performance of hemostatic agents, blood cell adhesion tests are commonly used [44]. In this study, we also used these tests to evaluate the hemostatic capacity of the hydrogel. We analyzed the rates of erythrocyte and platelet adsorption. Initially, we evaluated the hydrogel’s ability to adhere to red blood cells. As shown in Figure 5g, the hydrogel exhibited a significant adsorption force on red blood cells (RBCs), with no significant difference compared to the commercial adhesive (3M). The strong adhesiveness of the hydrogel may facilitate the adhesion of RBCs, while the porous network structure may contribute to the capture and aggregation of RBCs. Additionally, the LDH kit was used to measure the adherence of platelets to the hydrogel surface. As shown in Figure 5h, the hydrogel also exhibited a certain level of adhesion to platelets, with CT-5 demonstrating the highest adhesion strength, which was not significantly different from that of commercial adhesives. Investigations on blood clotting have demonstrated that hydrogel can enhance the formation of blood clots, the clustering of RBCs, and the attachment of platelets, promoting blood clotting as desired. It is hypothesized that hydrogels with strong adhesion can effectively enhance the adherence of RBCs and platelets. Additionally, a porous network structure can help trap and accumulate platelets. TA has a hemostatic activity that can significantly enhance the aggregation of red blood cells and platelets [45,46], thereby improving their coagulation ability [47]. TA can form strong hydrogen bonds and hydrophobic interactions with proteins on cell membranes [48]. Additionally, TA can accumulate Ca^2+^ due to the formation of complexes with metal ions, which plays a crucial role in the coagulation cascade.

After exploring the mechanical, adhesion, and coagulation properties of the hydrogels at different ratios, CT-5 was selected for further in vivo testing.

### 2.4. In Vivo Hemostatic Properties of CT-5

**Figure 6:**
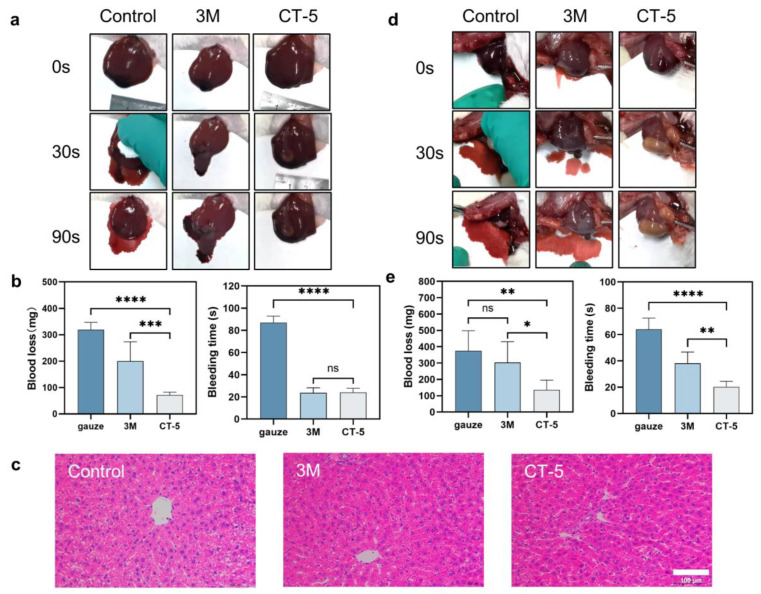
(**a**) Liver hemostasis test in rats; (**b**) blood loss and hemostasis time of the rat liver hemostasis experiment; (**c**) HE staining profile of rat liver on day 7; (**d**) cardiac hemostasis experiment in rats; (**e**) blood loss volume and hemostasis time of the rat cardiac hemostasis experiment. (scale bar: 100 μm, * *p* < 0.05, ** *p* < 0.01, *** *p* < 0.001, **** *p* < 0.0001, ns *p* > 0.05).

To demonstrate the potential of this hydrogel as a clinical hemostatic material, we applied it to hepatic and cardiac bleeding. First, the hemostatic property of the hydrogel was evaluated using the rat liver hemorrhage model, a specific visceral organ with abundant blood supply. As shown in Figure 6a, the model was created by cutting a thin piece of liver (10 mm × 2 mm). As shown in Figure 6b, the CT-5 group demonstrated a significant advantage in hemorrhage control with a total blood loss of 71.67 ± 10.60 mg, compared to the total blood losses of the commercial adhesive group (3M) and the gauze group, which were 199.83 ± 73.32 mg and 319.00 ± 27.77 mg, respectively. The hemostatic time of the CT-5 group and commercial adhesive groups was significantly shorter than that of the gauze group. However, there was no significant difference in the hemostasis time between the CT-5 group and the commercial group. (Figure 6b). In addition, as shown in Figure 6c, the hematoxylin and eosin (H&E) staining of liver tissues in rats after 7 days of liver bleeding indicated that CT-5 did not elicit an immune response or pathology, unlike the commercial adhesive group and the gauze group.

The hemostatic capacity and adhesion properties of the hydrogel were evaluated in a rat model of cardiac hemorrhage. The hydrogel must be able to withstand the burst pressure and mechanical disturbances caused by the beating heart [49]. As shown in Figure 6d, a bleeding wound was created in the heart area using a syringe needle to assess the sample’s hemostatic ability. Within the first 20 s of hemostasis, the CT-5 group tightly covered and bound to the heart injury site, preventing any further bleeding. In contrast, both the commercial adhesive group and the gauze group failed to stop the bleeding within 30 s. As shown in Figure 6e, the CT-5 group had significantly lower blood loss (135.83 ± 59.49 mg) compared to the commercial adhesive group (302.50 ± 128.97 mg) and the gauze group (374.83 ± 123.36 mg). The heart that was injured and treated with the hydrogel stopped bleeding immediately, confirming the hydrogel’s high efficacy in sealing severely bleeding tissues in a dynamic biological environment.

The strong tissue adhesion capacity of the hydrogel was responsible for its predominant hemostatic ability, despite the body’s deficiency in the coagulation mechanism [50]. Upon application to the bleeding site, the hydrogel acted as a physical barrier. On the other hand, collagen in hydrogels might activate platelets to promote blood clotting [28].

### 2.5. In Vivo Degradability of the CT-5

**Figure 7:**
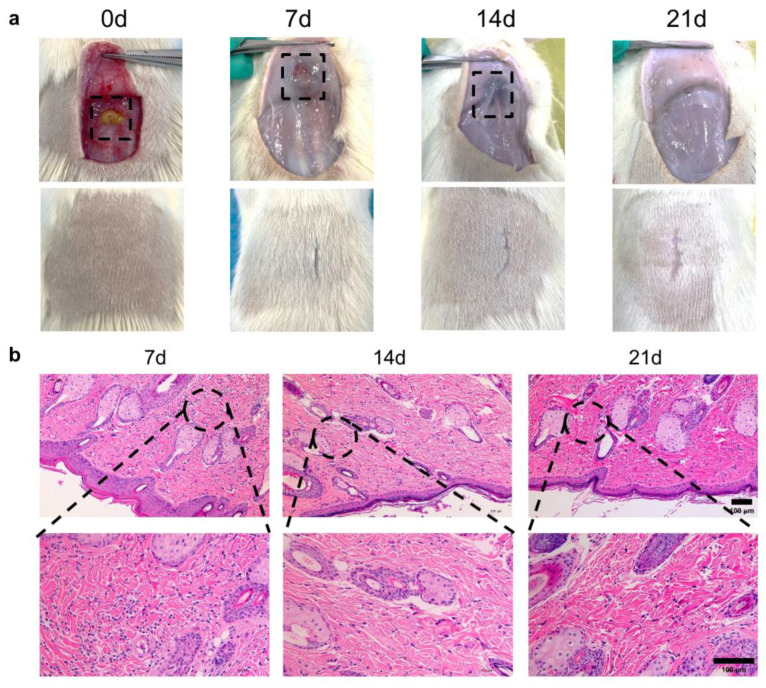
In vitro degradability. (**a**) Degradation of dorsal implantation in rats; (**b**) rat skin HE staining. (Scale bar: 100 μm).

A hemostatic hydrogel that is biodegradable can help in the regeneration of injured tissues. To assess hydrogel degradation and the host immune response in vivo, the hydrogel was implanted subcutaneously in rats. All rats remained healthy throughout the 3-week experiment and were sacrificed at 7, 14, and 21 days after the hydrogel implantation. The major organs, as well as the dorsal skin at the implantation site, were collected for subsequent experiments. As shown in Figure 7a, the subcutaneous implantation area of the hydrogel was observed on days 7, 14, and 21 with no visible signs of tissue necrosis, congestion, or pus. The volume of the hydrogel decreased significantly over time and was completely degraded by day 21, demonstrating its biodegradability in vivo. There was no redness or swelling on the surface of the implantation site on the rats’ backs, and the local fur appeared normal with no signs of fur loss.

Afterwards, we performed H&E staining to assess the host’s inflammatory response to the hydrogel. As shown in Figure 7b, after 7 days of implantation, the tissue surrounding the implantation site exhibited a visible inflammatory reaction with a minor lymphocyte infiltration, as observed microscopically, without congestion or necrosis. After 14 days of implantation, the inflammatory response decreased, and the number of inflammatory cells reduced. After 21 days of implantation, the inflammatory response was not significant, indicating a gradual weakening of the hydrogel’s inflammatory reaction. The amount of TNF-α in the skin of rats was measured, which also exhibited this phenomenon (Figure S8). Additionally, we examined the systemic toxicity of the hydrogel after 21 days, and no toxicity was detected in the major organs of rats (Figure S9). The results indicated that the subcutaneously implanted hydrogel causes only mild inflammatory responses in the host organism at initial stages and possesses good biodegradability.

Additionally, the blood biochemistry indexes, including aspartate aminotransferase (AST), alanine aminotransferase (ALT), and alkaline phosphatase (ALP) were further analyzed. As shown in Figure S10, there was no significant difference in the biochemical indexes of rats implanted with the hydrogel compared to the normal group, thus indicating normal liver and kidney function in the CT-5 group. Therefore, the hydrogel is biocompatible and does not affect the liver or kidney function of the body after degradation [51].

## 3. Conclusions

In this study, a hemostatic hydrogel with a strong adhesive effect was developed. Collagen and tannic acid were cross-linked by non-covalent bonds, and the hydrogel properties were concentration-dependent with respect to TA. In a humid environment, its adhesion strength could reach 90.99 ± 3.22 kPa, and it exhibited good procoagulant properties by rapidly recruiting RBCs and platelets, thus promoting the coagulation reaction. In the rat liver hemorrhage model, the blood loss was 71.67 ± 10.60 mg, and the bleeding time was 24 ± 3.74 s, which was significantly lower than that of the cyanoacrylate and gauze control group. The hydrogel degraded completely in vivo within 21 days and did not induce any toxicological, hepatic, or renal impairment in the organism. This study demonstrated that hemostatic hydrogel exhibits good biocompatibility, excellent mechanical properties, strong wet adhesion, and the ability to enrich blood cells and promote blood coagulation to achieve hemostasis. The major limitation of this study is that human blood was not used in the ex vivo experiments, which needs to be improved in subsequent work. Future studies should further validate whether this hydrogel activates the contact/intrinsic coagulation pathway through the activation of FXII. Considering the application of this hydrogel for human visceral hemostasis, subsequent experiments can be conducted using large animals such as dogs and pigs to verify the reliability of the results.

## 4. Materials and Methods

### 4.1. Materials

Collagen (collagen from chicken, type II) and tannic acid (TA) were obtained from Macklin Biochemical Technology Co., Ltd. (Shanghai, China). Triton X-100 was acquired from Sigma-Aldrich Inc. (Louis, MO, USA). An LDH kit was obtained from CAYMAN chemical company (Ann Arbor, MI, USA). Commercial adhesive (cyanoacrylate) was obtained from 3M (Japan). Isopropanol was obtained from Sinopharm (Beijing, China). Phosphate buffered saline (PBS) was obtained from Pricella (Wuhan, China). A TNF-α Elisa kit was obtained from Solarbio (Beijing, China). All chemicals were received and used without further purification. Sprague–Dawley (SD) rats were obtained from the Animal Resource Center of the National Institute for Food and Drug Control, with experimental animal use permission No. SYXK (Beijing) 2022-0014.

### 4.2. Synthesis of Hydrogels

First, different concentrations of tannic acid solution and 10 wt% collagen solution with deionized water were prepared. Then, the TA solution was added to the collagen solution and stirred well. The mass ratios of tannic acid and collagen in the binder were 1:1, 1:2, 1:3, 1:4, and 1:5, respectively, and were labeled CT-1, CT-2, CT-3, CT-4, and CT-5, respectively.

### 4.3. Characterization of Hydrogels

The TA, collagen, or CT were freeze-dried into a powder, mixed with potassium bromide, and formed into thin disks. Then, the FTIR spectrum of TA, collagen, or CT was obtained with a Fourier transform infrared spectrometer (Nicolet iS 10, Thermo Scientific, Waltham, MA, USA) in the 4000–400 cm^−1^.

The interfaces of hydrogels were examined by SEM (JSM-7800F, JEOL, Tokyo, Japan). Hydrogels were applied to the conductive adhesive and observed with SEM.

### 4.4. Swelling Ratio Test

The hydrogel was weighed (recorded as *W*_0_) and soaked in PBS (pH = 7.2) at room temperature. At regular intervals, the hydrogel was taken out, and water on the surface was wiped off and weighed (recorded as *W*_1_). The swelling ratio (%) of hydrogel was calculated from:

$$\mathrm{Swelling}\;\mathrm{ratio}\;\left(\%\right)=\frac{W_{1}-W_{0}}{W_{0}}\times 100\%$$

### 4.5. Rheological Analysis

The hydrogels were evaluated through rheological tests using a rotary rheometer (HR10, New Castle, DE, USA) with frequency sweep tests ranging from 0.1 to 100 rad/s at a constant strain of 0.1% (37 °C) to monitor changes in the storage modulus and loss modulus. The shear rate was set to 0.1–100 S^−1^, and flow scanning was performed to measure the viscosity of the sample.

### 4.6. In Vitro Degradation of Hydrogels

The hydrogel was weighed (recorded as *M*_0_) and soaked in PBS (pH = 7.2) at a 37-water bath shake. At regular intervals, the hydrogel was taken out, and water on the surface was wiped off and weighed (recorded as *M*_1_). The degradation ratio (%) of hydrogel was calculated from:

$$\mathrm{Degradation}\;\mathrm{ratio}\;\left(\%\right)=\frac{M_{0}-M_{1}}{M_{0}}\times 100\%$$

### 4.7. Adhesion Strength Test

According to ASTM protocol, the adhesion strength of hydrogels was tested. The oil was removed from the fresh porcine skin (40 mm × 10 mm × 2 mm) with isopropanol. The hydrogel was placed between the two pieces of porcine skin and pressed for an adequate amount of time. Finally, they were fixed on a universal materials tester (HKE-3511H, Dongguan, China) for lap shear strength, tensile strength, peel strength, and wound-closure strength with a strain rate of 5 mm/min. In the same way, other substrates were made into strips and firmly bonded with the hydrogel, after which shear adhesion tests were performed.

### 4.8. Wet Bond Strength

Referring to Section 4.7, the hydrogel was adhered to the pig skin, soaked in phosphate buffered saline (PBS) (pH = 7.2), and the shear adhesion, tensile bonding, peeling experiment, and wound closure strength test of the hydrogel paste on the porcine skin were carried out, respectively.

### 4.9. Water Contact Angle

A contact angle tester was used to test the water contact angle of the hydrogel. The samples were evaluated by making a cylindrical sample (20 mm × 2 mm), adding 10 μL of water droplets to the surface of the sample to stabilize it, and measuring the water contact angle using a contact angle tester.

### 4.10. Burst Pressure Test

According to the ASTM F2392, a burst pressure test was performed. After removing excess fat from the porcine skin (2.5 mm × 2.5 mm), holes (3 mm) were punched in the center. The skin was then attached to the mold, which was linked to a syringe pump. The hydrogel was then applied to the incision site to determine its maximum burst pressure.

### 4.11. Hemolysis Ratios

Citrate whole blood (CWB) was obtained from a rabbit by mixing the blood with anticoagulant citrate dextrose (9:1). Normal saline (4:5) was added to the CWB to obtain freshly diluted anticoagulated rabbit blood. The hydrogel was extracted with normal saline at a ratio of 0.2 g/mL. Normal saline was used as a negative control, pure water was used as a positive group, and the temperature of each group was kept at 37 °C for 30 min, then 0.2 mL of diluted rabbit blood was added and shaken. After that, it was put in a water bath at 37 °C for 60 min and centrifugation was carried out at 2000 r/min for 5 min. The supernatant was transferred to a cuvette, and the *OD* value of each group was measured at 545 nm. The hemolysis rate was calculated by the formula:

$$\mathrm{Hemolysis}\;\mathrm{Ratios}\;\left(\%\right)=\frac{\left(OD_{sample}-OD_{negative}\right)}{\left(OD_{positive}-OD_{negative}\right)}\times 100\%$$

### 4.12. Blood Clotting Index

For the blood clotting index (BCI) assay, the hydrogels were put in 24-well plates. Then, 10 μL of CWB was dropped into each group. Next, 2 μL of 0.1 M CaCl_2_ was added in sequence to activate the coagulation cascade in the with CaCl_2_ model, while the model without CaCl_2_ proceeded directly to the next step without adding CaCl_2_. The plates were kept at 37 °C for 5 min, then 2 mL of pure water was added to lyse the uncoagulated erythrocyte with further shaking for 10 min. The *OD* value of the solutions was measured at 540 nm and recorded as A*_S_*. The citrate whole blood activated by Ca^2+^ without hydrogel was used as a reference and the *OD* value was recorded as A*_B_*. BCI was calculated by the formula:

$$\mathrm{BCI}\;\left(\%\right)=\frac{A_{S}}{A_{B}}\times 100\%$$

### 4.13. Attachment of Red Blood Cells

Hydrogels were put in 24-well plates, then 50 μL CWB was dropped into each group, incubated at 37 °C for 10 min, and then the non-adhered red blood cells (RBCs) were washed with PBS. Then, 1 mL Triton X-100 (1%) was added onto the hydrogels incubated at 37 °C for 1 h to lyse the adherent RBCs. The *OD* value of the solutions was measured at 540 nm, and 50 μL CWB in 1 mL Triton X-100 (1%) was used as the reference. The attachment of RBC was calculated by the formula:

$$\mathrm{Attachment}\;\mathrm{of}\;\mathrm{RBC}\;\left(\%\right)=\frac{OD_{sample}}{OD_{reference}}\times 100\%$$

### 4.14. Attachment of Platelets

Firstly, 10 mL of CWB was centrifuged at 1000 rpm for 10 min. Then, 2 mL of supernatant was removed and then centrifuged at 2000 rpm for 10 min to obtain platelet-rich plasma (PRP). Hydrogels were put in 24-well plates, then 50 μL PRP was dropped into each group and incubated at 37 °C for 10 min. Then, the non-adhered platelets were washed with PBS. Next, 300 μL Triton X-100 (1%) was added onto the hydrogels incubated at 37 °C for 1 h to lyse the adhered platelets. The absorbance of the solutions was measured at 490 nm by ultraviolet measurement according to the LDH kit. A total of 50 μL PRP without treatment was used as the reference. The attachment of platelets was calculated by the formula:

$$\mathrm{Attachment}\;\mathrm{of}\;\mathrm{platelets}\;\left(\%\right)=\frac{OD_{sample}}{OD_{reference}}\times 100\%$$

### 4.15. Hemostasis on Rats’ Liver and Heart

Eighteen SD rats of both sexes (200–250 g) were used. For the hemostasis of the liver, an incision (10 mm × 2 mm) was made on the liver surface with a blade. The hydrogel was placed on the bleeding wound. Cyanoacrylate (3M) and gauze were used as controls. The blood from the liver wound was collected with filter paper. The blood loss and the bleeding time were recorded. After hemostasis, the wound was sutured; the rats were euthanized after 7 days of continuous feeding, and the liver tissue from the hemostasis site was taken for HE staining.

For the hemostasis of the cardiac model, a needle with a diameter of 1.2 mm was used to pierce the heart. The wounds were immediately coated with the hydrogel in the CT-5 group while they were covered with gauze or cyanoacrylate (3M) in the control group. The blood from the wound was collected with filter paper. The blood loss and the bleeding time were recorded.

### 4.16. In Vivo Degradation of Hydrogel

To assess the tissue compatibility and in vivo degradation capacity of the hydrogel, the hydrogel was implanted subcutaneously in the back of rats. Before surgery, hydrogels were sterilized with ultraviolet light. Nine SD rats (150–200 g, male) were anesthetized with pentobarbital and the back hair was shaved. A long incision (2 mm) was made with surgical scissors on the back of the rat and the hydrogel was implanted into it. After 7, 14, or 21 days, the rats were necropsied to observe the skin and hydrogel degradation at the implantation sites, and the skin at the implantation sites and the major organs of the rats were collected for hematoxylin-eosin (H&E) staining and determination of the TNF-α content in the skin. The H&E staining images were recorded with a DM IL LED microscope (Leica, Wetzlar, Germany).

### 4.17. Statistical Analysis

All experiments were independently repeated at least three times (*n* ≥ 3) and presented as mean ± standard deviation. Statistical significance between the results was determined by one-way ANOVA and Student’s *t*-test. *p* < 0.05 was considered to be statistically significant (* *p* < 0.05, ** *p* < 0.01, *** *p* < 0.001, **** *p* < 0.0001).

## Data Availability

The data presented in this study are available in this article.

## Supplementary Materials

The following supporting information can be downloaded at: https://www.mdpi.com/article/10.3390/gels10050326/s1, Figure S1: Structure of tannic acid; Figure S2: FTIP of the hydrogel in the wet state; Figure S3: The G′ and G′′ of hydrogels; Figure S4: SEM of the hydrogel; Figure S5: Tensile strength of common substrates; Figure S6: Tensile shear and peel strength; Figure S7: Adhesion capacity of major organs; Figure S8: TNF-α content in vivo degradation assays; Figure S9: Pathological sections of major organs for in vivo degradation experiments. scale bar: 100 μm; Figure S10: Blood biochemistry analysis of ALT, AST, and ALP.
