# Supplementary material for: Non-Covalent Cross-Linking Hydrogel: A New Method for Visceral Hemostasis
Source: Gels. 2024 May 10;10(5):326. doi: 10.3390/gels10050326 (PMC11121205; doi:10.3390/gels10050326)
Supplement: Supplementary file 1 [file gels-10-00326-s001.zip › gels-2945949-supplementary.pdf]

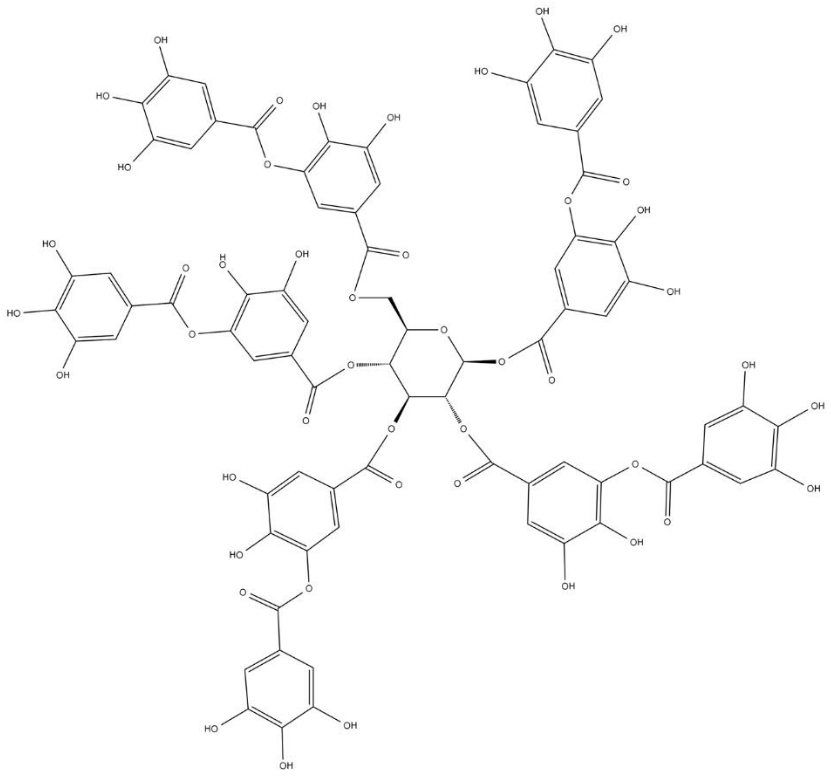

Figure S1．Structure of tannic acid

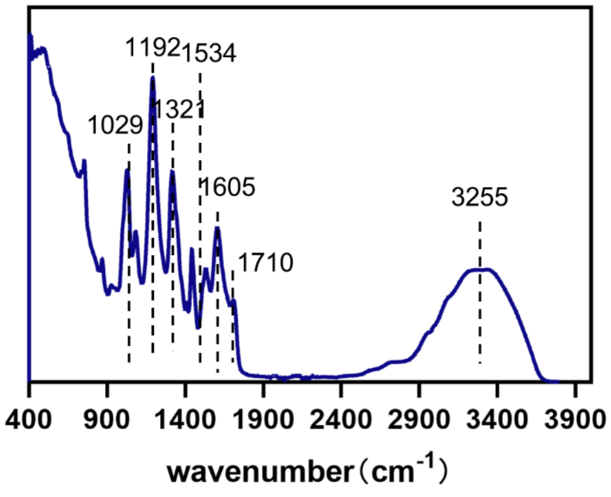

Figure S2．FTIP of the hydrogel in the wet state

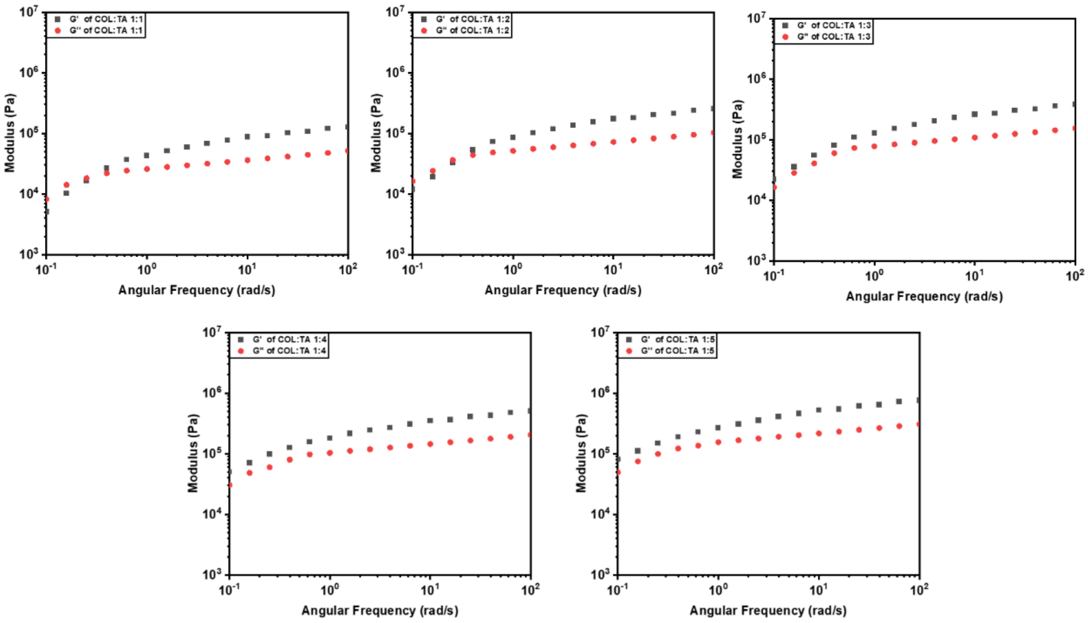

Figure S3． The G′ and G″ of hydrogels

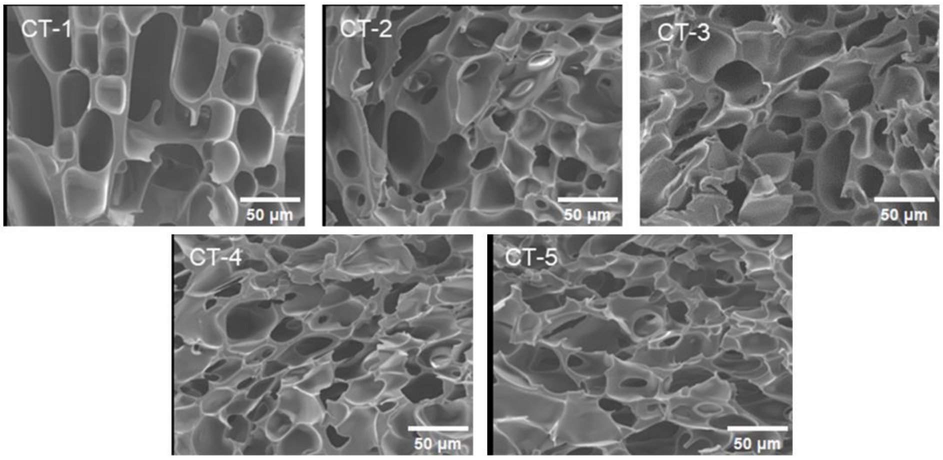

Figure S4． SEM of the hydrogel

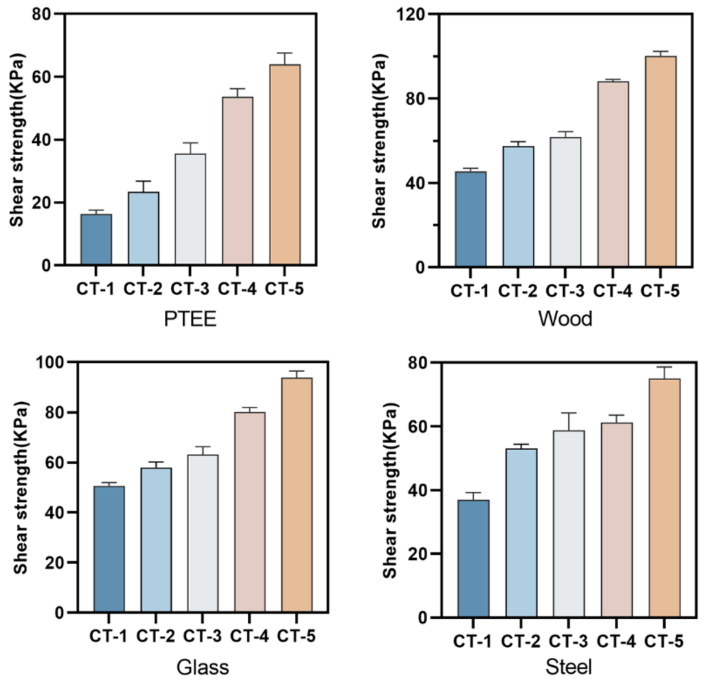

Figure S5. Tensile strength of common substrates

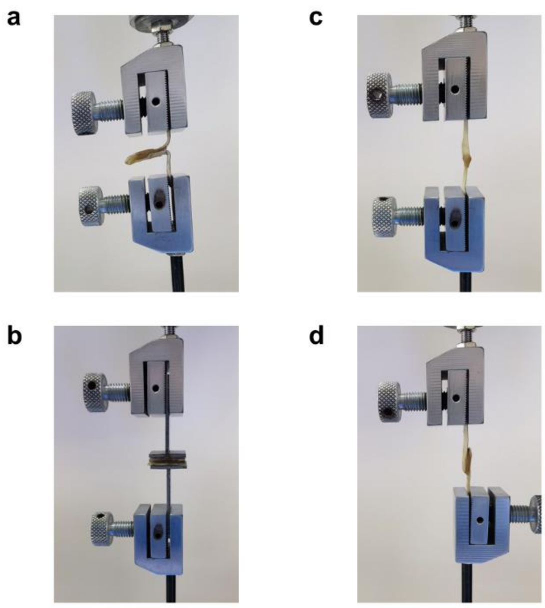

Figure S6. Tensile shear and peel strength.

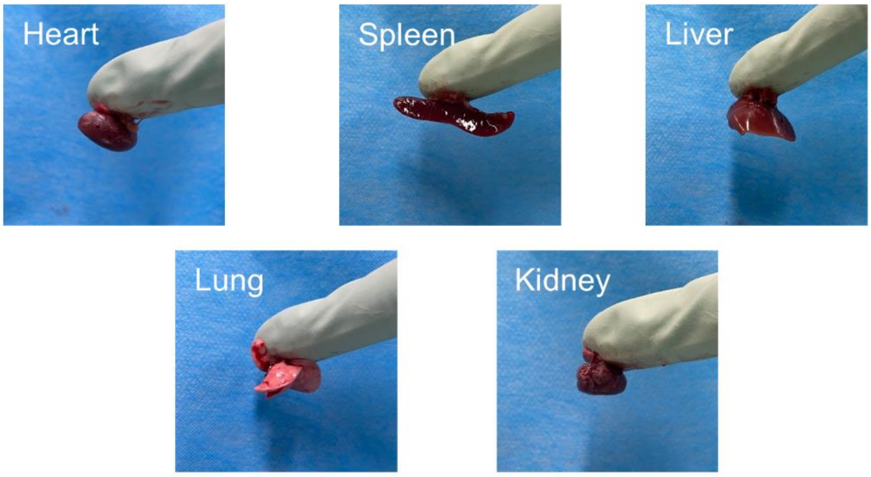

Figure S7． Adhesion capacity of major organs.

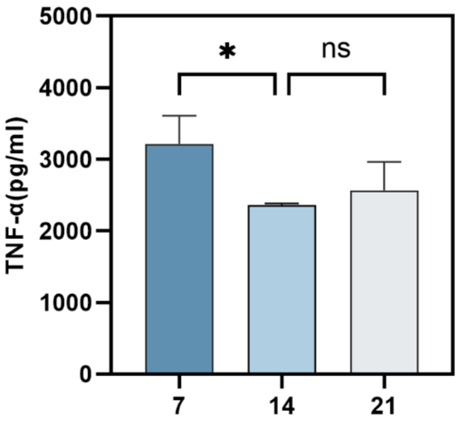

Figure S8． TNF-α content in vivo degradation assays

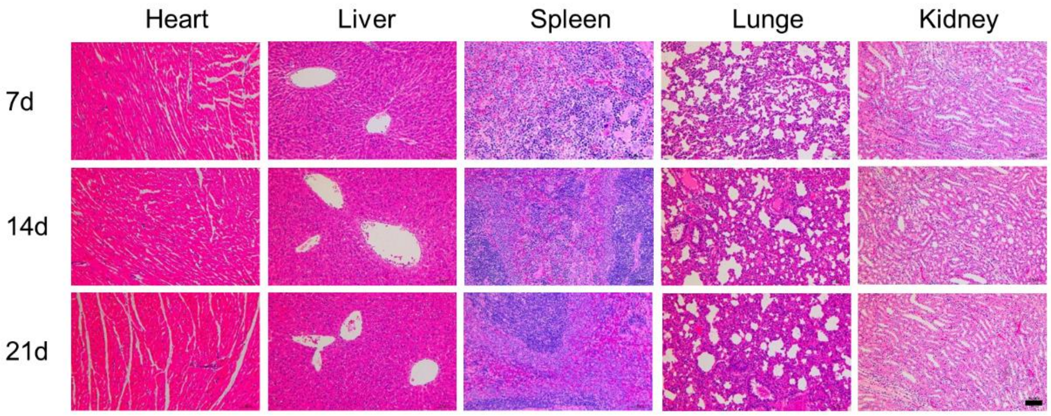

Figure S9． Pathological sections of major organs for in vivo degradation experiments. scale bar: 100μm

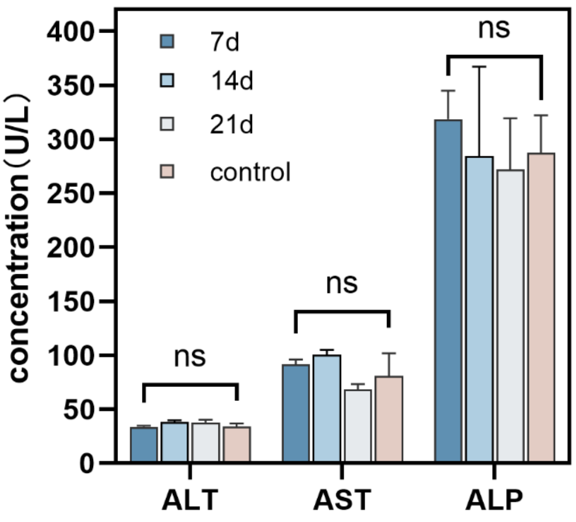

Figure S10． Blood biochemistry analysis of ALT, AST, and ALP.
